# Phloem cell responses to the feeding activity of *Eriosoma lanigerum* on *Malus domestica*

**DOI:** 10.3389/fpls.2025.1507552

**Published:** 2025-01-30

**Authors:** Ravena Malheiros Nogueira, Gracielle Pimenta Pereira Bragança, Edgard Augusto de Toledo Picoli, Denis Coelho de Oliveira, Rosy Mary dos Santos Isaias

**Affiliations:** ^1^ Department of Botany, Universidade Federal de Minas Gerais, Belo Horizonte, Minas Gerais, Brazil; ^2^ Department of Plant Biology, Universidade Federal de Viçosa, Viçosa, Minas Gerais, Brazil; ^3^ Institute of Biology, Universidade Federal de Uberlândia, Uberlândia, Minas Gerais, Brazil

**Keywords:** aphid galls, cytology, plant tumor, plant-insect interactions, secondary phloem, sieve tube element

## Abstract

**Introduction:**

Hemipteran gall vascular traits result from the access, piercing, and sucking of the inducer mouth parts directly in the xylem and phloem conductive cells. Herein, our focus relies on mapping the features of phloem cells in the proximal, median, and distal regions of *Malus pumila* stem galls and adjacent galled stems.

**Methods:**

Phloem cells were dissociated from gall fragments, the stem portions above and below the galls, and the proximal and distal regions of *M. pumila* stem galls. were measured. The comparison of the higher length and diameter of the sieve tube elements (STE) was evaluated considering the priority of nutrient flow to gall portions.

**Results:**

In the *M. pumila - E. lanigerum* system, there were no significant differences in the dimensions of the STE in the galls compared with those of the stem portions above and below the galls.

**Discussion:**

At the cytological level, the callose deposited in gall STE and the decrease in the cell lumen area in the stem portion above the gall due to thickened nacreous cell walls have implications for nutrient flow. Peculiarly, the smaller sieve pores in the sieve plates of the STE located in the galls and stem portions above and below them and the deposition of P-protein in the stem portions below the galls limit the bidirectional transport of nutrients, benefiting the transport of photoassimilates to the gall proximal region and reducing the vigor of apple tree stems.

## Introduction

The feeding behavior of distinct taxa of galling organisms may induce neoformations in their host plant tissues (Mani, 1964; Bronner, 1992; Ferreira et al., 2017; Álvarez et al., 2021). Some galling insects, such as Psylloidea and Aphididae, have long stylets that are inserted inside phloem cells, their primary feeding site (Wool, 2005; Álvarez, 2012; Álvarez et al., 2014; Muñoz-Viveros et al., 2014; Álvarez et al., 2016). The impact of these stylets on phloem cells is reported to be mainly cell hypertrophy (Álvarez et al., 2016; Ferreira et al., 2017, 2019; Álvarez et al., 2020; Martini et al., 2020), which is a facilitating factor for inducers by increasing the area of sap transportation.

The transport of nutrients from the source to the sink organs through sieve tubes is elementary, and the sequences of sieve tube elements (STE) connected by sieve plates in their end walls form a contiguous transport pathway (Lalonde et al., 2003; van Bel, 2003). The long-distance transport of photoassimilates and other signaling molecules, such as sugars, amino acids, and hormones, occurs through STE that lose their nuclei during maturation (Esau and Cronshaw, 1968; Knoblauch and Peters, 2013; Savage et al., 2016; Aloni, 2021). These substances are translocated from the producing leaves to developing young tissues at the plant tips and to storage organs (Yan and Liu, 2020) and, accordingly, to gall developmental sites that function as additional sinks. Phloem translocation is bidirectional, the water flows from a region of high turgor pressure towards a region of low turgor pressure, meaning the flow of food is in both directions, both upwards and downwards, depending on the gradient formed (White and Ding, 2023). According to Münch’s hypothesis (Münch, 1930), the mass flow occurs due to a pressure gradient, generated osmotically between the source and the drain. The active loading of sugars generates a low osmotic potential in the sieve tube elements of the source, and consequently the inflow of water results in an increase of turgor (Patrick, 1997). In the drain, unloading leads to a reduction in water potential in the sieve tubes and sugars are released at low pressure into the drains (Hafke et al., 2005).

In contrast, the alterations generated by gall induction in plant vascular system may lead to a prioritization of water transport by the vessel elements explained by the gall constriction hypothesis proposed by Aloni et al. (1995). This hypothesis predicts that the stem regions below the gall have greater xylem differentiation with rays and vessel elements of regular size, whereas the region above the gall has narrower vessel elements, enlarged rays and an absence of fibers, which limits water transport to the aerial part of the plant (Aloni et al., 1995).

The infestation by *Eriosoma lanigerum* (Aphididae), a phloem feeder in the stems and roots of *Malus domestica* cv. ‘Eva’ (Brown and Schmitt, 1990), induces abnormal vascular cambium activity with high proliferation of vascular parenchyma cells and neoformation of vessel elements, both in the roots and stems of apple trees (Freitas, 2021). The galls may be divided into three regions according to the position of the *E. lanigerum* colonies on the host organ surface: proximal, median, and distal. The proximal region has islets of secondary vascular parenchyma cells with interspersed conductive elements. The median region has hyperplasic parenchyma and grouped tracheary elements. The distal region is located at the opposite site of the *E. lanigerum* colonies, and its anatomical organization is similar to that of the nongalled stems (Freitas, 2021). This gall infestation can result in reduced tree vigor due to the redirection of photoassimilates to *E. lanigerum* colony support (Madsen and Bailey, 1958; Ateyyat and Al-Antary, 2009), leading to the death of infested *M. domestica* plants (Ateyyat and Al-Antary, 2009).

As gall-inducing phloem feeders may modify the structure of their host plant phloem (Brown and Schmitt, 1990; Richardson et al., 2016; Álvarez et al., 2021), facilitating access to nutritive resources, we hypothesize that the STE within the galls may be larger than those of the non-galled host organs of the apple trees. In parallel with the hypothesis of vascular constriction proposed for the xylem (Aloni et al., 1995), we expect the STE to be smaller in the region above the galls, forcing a gradient pressure and prioritizing the transport of photoassimilates to the galled regions. To test this hypothesis, we investigated the phloem features of the stem galls induced by *E. lanigerum* on *M. domestica* and discussed the functional implications for gall development.

## Material and methods

Samples of non-galled stems and stem galls were collected from *M. domestica* individuals (cultivar ‘Eva’) (n ≥ 5, per category) in a private commercial orchard in the municipality of Ervália, Minas Gerais, Brazil (20°52’02’’S, 42°38’41’’W). For anatomical analyses, the samples were fixed in Karnovsky’s solution (2.5% glutaraldehyde and 4.5% formaldehyde) (Karnovsky, 1965, modified to 0.1 M phosphate buffer, pH 7.2) for 48 h, dehydrated in an ethanol series, and embedded in 2-hydroxyethyl methacrylate (Historesin, Leica^®^ Instruments, Germany). The fragments (1 cm²), comprising regions with phloem, were divided into five samples: (1) non-galled stem branches, (2) the gall proximal region, (3) the gall distal region, (4) the portion below the galls, and (5) the portion above the galls (n = 5 per category). These fragments were sectioned in two anatomical planes (transverse and tangential longitudinal). The sections (6 μm) were obtained with a rotatory microtome (Leica^®^ 97 2035 BIOCUT) and stained with 0.05% toluidine blue O, pH 4.7 (O’Brien et al., 1964). The slides were mounted in Entellan (Kraus and Arduin, 1997) and analyzed under a photomicroscope (Leica^®^ DM500 Wetzlar, Germany) with a coupled digital camera (Leica^®^ ICC50HP; Wetzlar, Germany). For callose, the sections were stained with 0.1% aniline blue for 30 min (Johansen, 1940). The material was washed, mounted in water, and photographed under a fluorescence microscope (Leica^®^ DM2500LED) with a DAPI filter (330–385 nm) and emission light (420 nm) and a coupled digital camera (Leica^®^ DFC 7000T).

For cytological analysis, fixed fragments (1 cm²) of the five samples (n = 5 per category) were postfixed in 1% osmium tetroxide (OsO4) in 0.1 mol/L PBS, dehydrated in an ethanol series (O’Brien and McCully, 1981), and embedded in Spurr^®^ resin (Sigma-Aldrich) (Luft, 1961). The samples were sectioned in two anatomical planes (transverse and tangential longitudinal) in an ultramicrotome, Reichert-Jung Ultracut (Leica, Wetzlar, Germany), contrasted with uranyl acetate and lead citrate (Reynolds, 1963), and analyzed under a transmission electron microscope, Tecnai™ G2-12 Spirit BioTwin (FEI, Hillsboro, USA; 120 KV), at the Centro de Microscopia of the Universidade Federal de Minas Gerais (CM-UFMG).

For statistical analyses, the number of STE in an image were counted in transverse sections of nongalled stem (NG), gall distal (DR) and proximal regions (PR) of n = 5 individuals, 10 images per individual, total of 50 STE per individual). Parametric data of the mean width of STE in nongalled stem (NG), gall distal (DR) and proximal regions (PR) were compared via one-way ANOVA (for three or more categories) followed by Tukey’s test. Nonparametric data were compared with the Mann-Whitney test (for two categories), and and Kruskal-Wallis test (for three or more categories). The tests were performed with SigmaStat^®^ (Systat Software, Inc., Chicago, Illinois), and the graphs were generated with GraphPad Prism 8.0^®^ software. All tests used α = 0.05.

## Results

**Figure 1:**
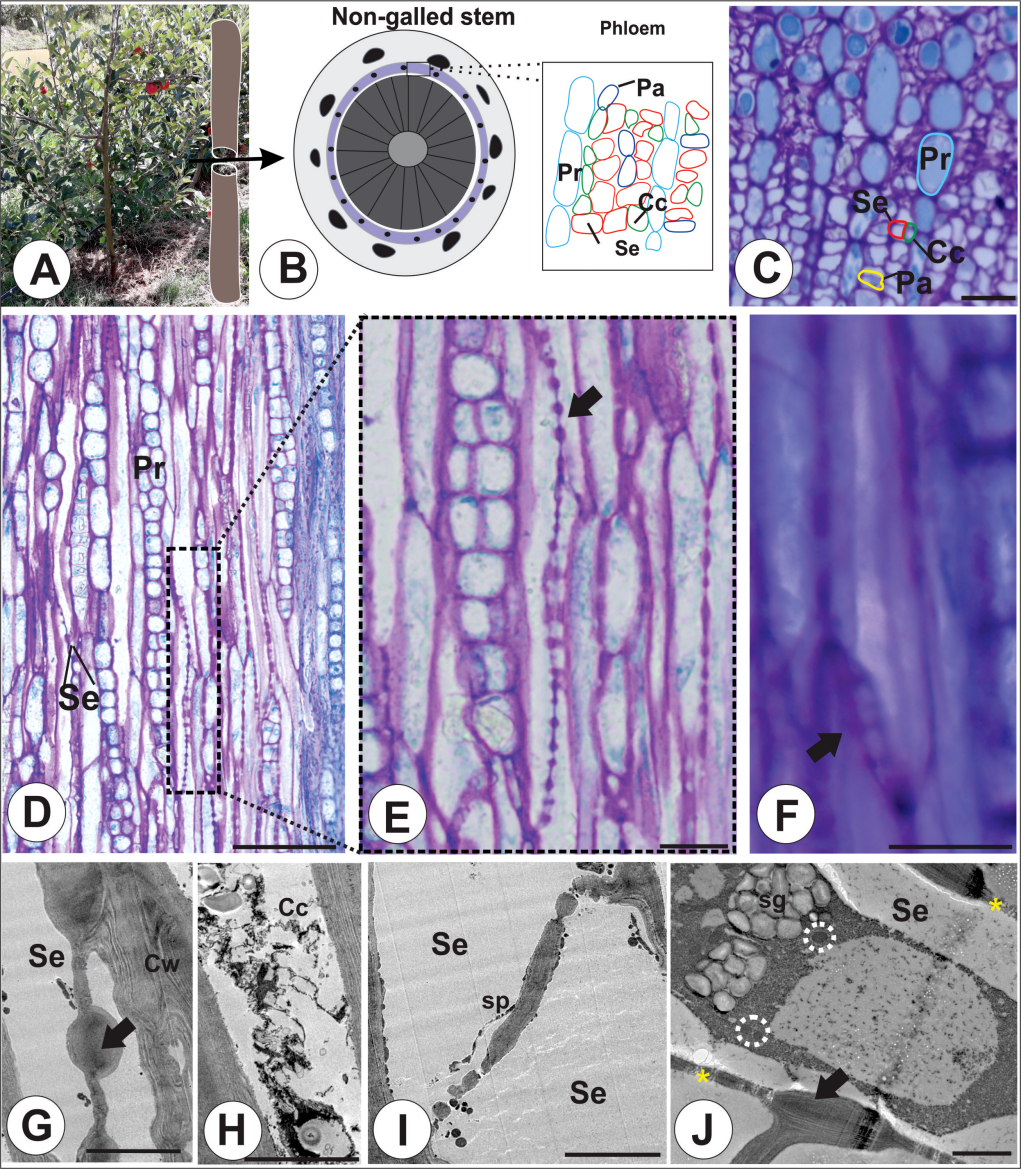
Non-galled stems of *M. domestica*. **(A)** Apple tree. **(B)** Diagram of a non-galled stem with highlighted phloem cells. **(B, C)** Transverse section of the phloem showing STE (red circle), dense companion cells (green circle), vascular parenchyma (dark blue circle), and parenchymatic rays (light blue circle). **(D, F)** Longitudinal sections. **(D)** Phloem with parenchymatic rays and STE and sieve areas on the sidewalls (dotted rectangle). **(E)** Lateral sieve areas (black arrow; dotted square). **(F)** Details of the inclined sieve plate (black arrow). **(G, I)** Transmission electron micrographs. **(G)** Lateral sieve area (black arrow) in a sieve tube element. **(H)** Sieve plate on a terminal wall. **(I)** Plasmodesmata connections (yellow asterisks) between sieve areas (black arrow) and juvenile protoplasts, as evidenced by starch grains and mitochondria (dotted circles). Cc, companion cells; Cw, cell wall; Pa, parenchyma; Pr, parenchymatic ray; Se, sieve tube elements; Sp, sieve plate; Sg, starch grains. Bars: **(C, F)** 50 μm. **(G, H)** 5 μm. **(I)** 2 μm.

The phloem of the nongalled stems of *M. domestica* (**Figures 1A, B**) is composed of STE with irregular contours associated with small companion cells with dense cytoplasm (**Figure 1C**) and parenchyma cells adjacent to the complex of companion cells and STE (**Figure 1D**). In tangential longitudinal sections, the secondary phloem is composed of biseriate rays with parenchyma cells and long, continuous rows of STE with multiple sieve areas on the sidewalls (**Figures 1D, E**). Composite sieve plates are observed in oblique end walls (**Figure 1F**), and large circular sieve areas are observed in the sidewalls (**Figure 1G**). The companion cell contains cytoplasm with vesicles and starch grains (**Figure 1H**). The sieve plates can be either transverse or inclined, and the sidewalls have sieve areas unobstructed by callose (**Figure 1I**). Starch grains accumulate in the plastids, and mitochondria are observed in the young sieve tube elements, which have conspicuous plasmodesmata connections and elongated sieve areas (**Figure 1J**).

**Figure 2:**
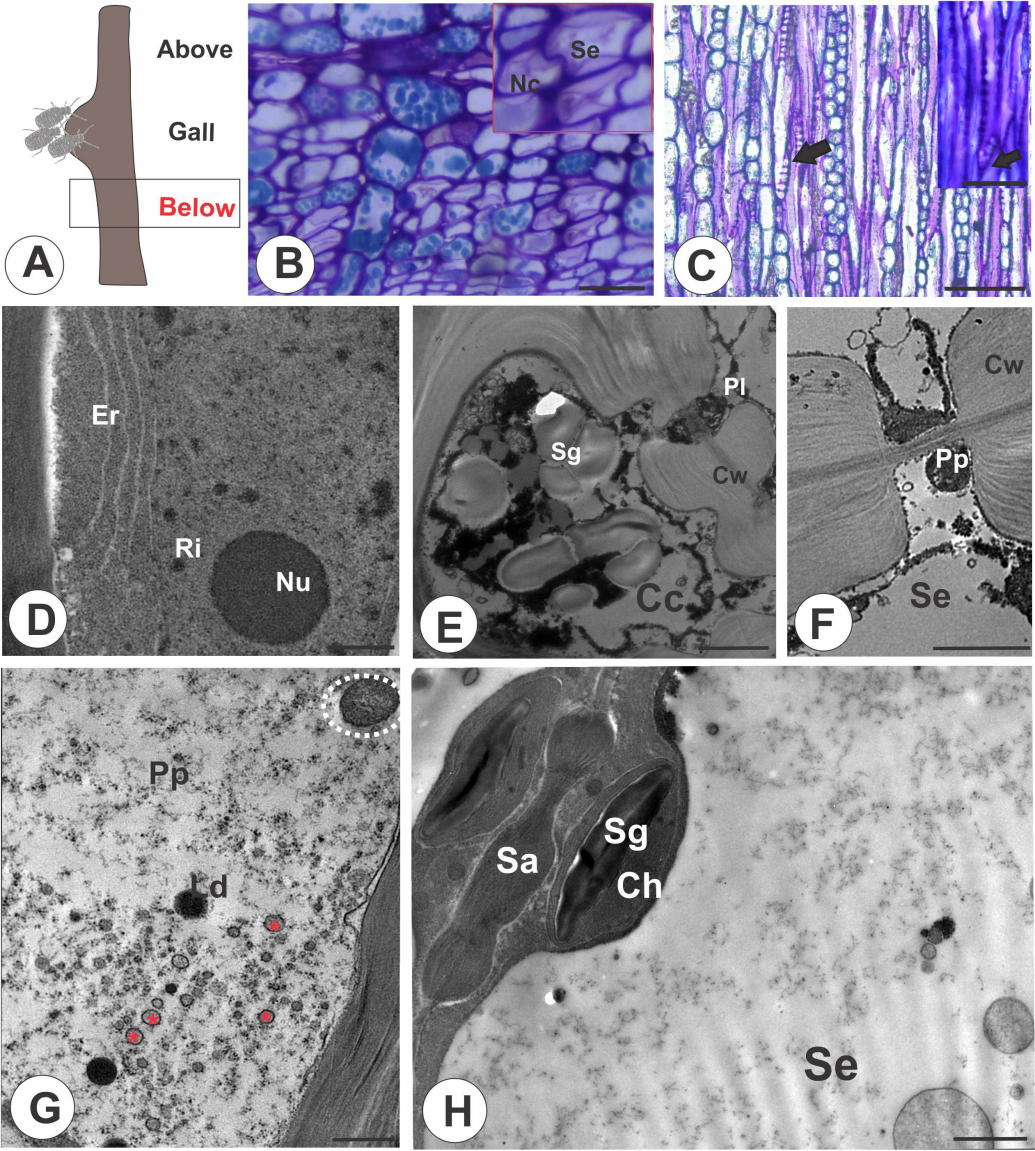
Stem portion below the galls on *M. domestica.* **(A)** Schematic representation of a stem gall showing the portion below the gall induced by a colony of *E lanigerum*. **(B)** Transverse section of phloem showing the nacreous walls of STE (red square). **(C)** Longitudinal section of a terminal sieve plate in detail (black arrow). **(D, H)** Transmission electron micrographs. **(D)** Ordinary companion cell with evident nucleus and nucleolus, endoplasmic reticulum, and ribosomes. **(E)** Intermediate companion cell with a thick undulated cell wall, protoplast with vesicles, and starch grains. A P-type plastid can be seen in a sieve tube element. **(F)** Details of a P-protein in a sieve tube element. **(G)** STE with dispersed filamentous P-protein, mitochondria (white dotted circle), lipid droplets, and lamellar bodies (red asterisk). **(H)** STE associated with a companion cell through a sieve area; chloroplasts with starch are observed. Cc, companion cells; Ch, chloroplast; Cw, cell wall; Er, endoplasmic reticulum; Se, sieve tube elements; Sa, sieve area; Sg, starch grains; Nc, nacreous walls; Ls, lipophilic substance; Nu, nucleolus; Pp, P-protein; P-p, P-type plastid; Ri, ribosome. Bars: **(B, C)** 50 μm; **(D, E)** 2 μm; **(F, G)** 1 μm**; (H, I)** 2 μm.

In the stem portions below the galls (**Figure 2A**), nacreous walls (**Figure 2B**) and oblique sieve plates in STE (**Figure 2C**) are evident. The ordinary companion cells contain a dense cytoplasm, an elongated nucleus, nucleolus, ribosomes, and the endoplasmic reticulum (**Figure 2D**). In the intermediate type of companion cell, the walls are undulate and contain cytoplasm with vesicles and starch grains (**Figure 2E**). The STE contain cytoplasm with granular and filamentous P-protein, lipid droplets, lamellar bodies (**Figures 2F, G**), and mitochondria; starch grains accumulate in the chloroplasts (**Figure 2H**).

**Figure 3:**
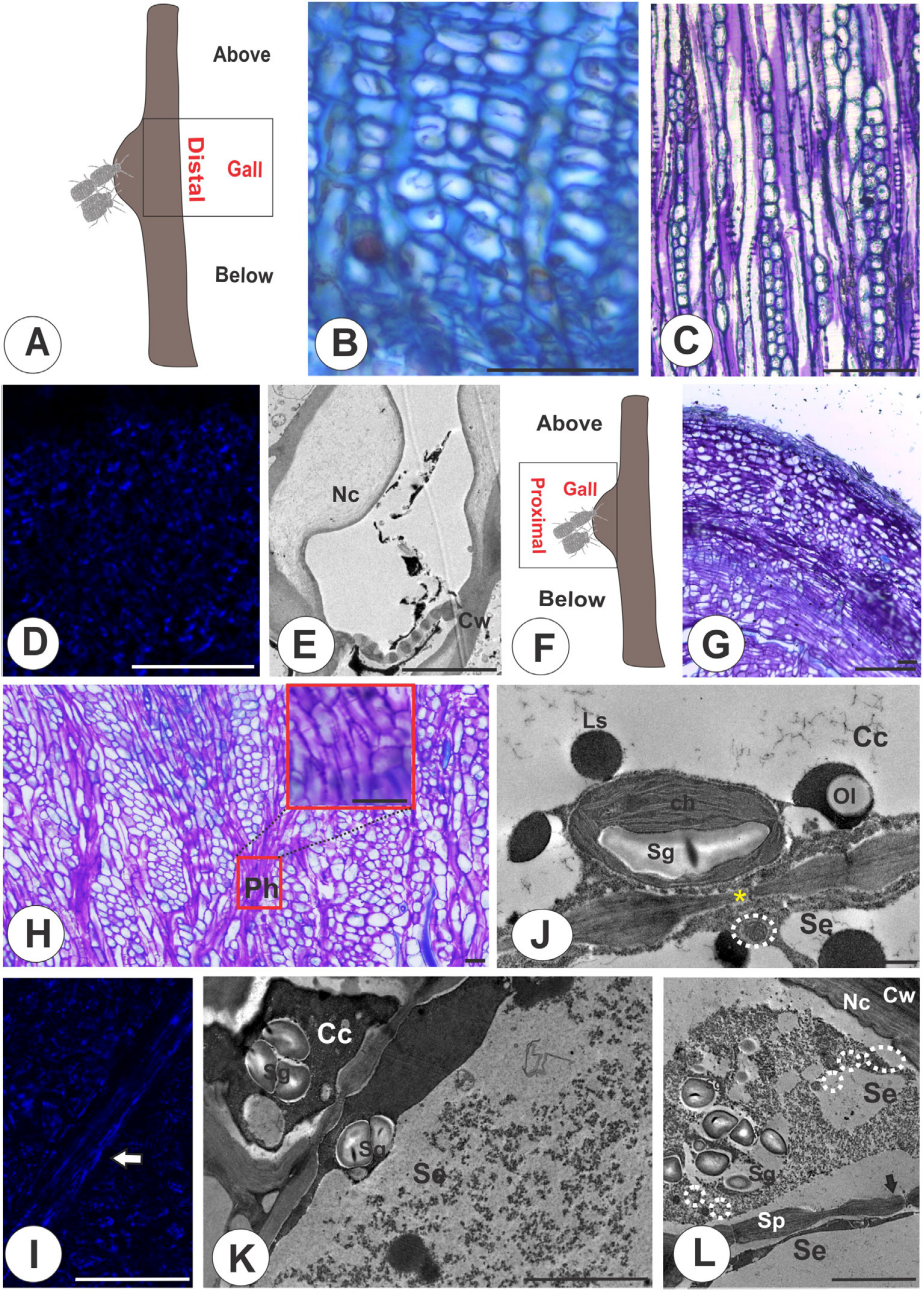
Distal and proximal regions of galls on *M. domestica*. **(A)** Schematic representation of a stem gall showing the region of the gall distal to the colony of *E lanigerum.* **(B, E)** Distal region. **(B)** Transverse section of the phloem in light microscopy. **(C)** Tangential longitudinal section of the phloem. **(D)** Callose detection with aniline blue under fluorescence and a DAPI filter. **(E)** Transmission electron micrograph (TEM) showing nacreous walls and callose in sieve plates. **(F, I)** Proximal region. **(F)** Schematic representation of a stem gall evidencing the proximal region of the gall. **(G)** Tangential longitudinal section of the parenchyma cells interspersed with the sieve elements in the proximal region, which lose their axiality. **(H)** Tangential longitudinal section of the STE connected by sieve plates. **(I)** Callose detection with aniline blue under fluorescence and a DAPI filter (white arrow). **(J, L)** TEM. **(J)** Sieve area connecting plasmodesmata (yellow asterisk) and chloroplasts with associated starch grains. **(K)** Starch in chloroplasts and lipid droplets in companion cells. **(L)** STE with numerous mitochondria and accumulated starch grains. Arrows indicate the sieve areas. Cc, companion cells; Ch, chloroplasts; Cw, cell walls; Nc, nacreous; Se, sieve tube elements; Sg, starch grains; Sp, sieve plate; Ls, lipophilic substance; Ol, oleosome. Bars: **(B, D, G, I)** 50 μm; **(E; K, I)** 5 μm; **(J)** 1 μm.

The sieve tubes in the distal region of the galls (**Figure 3A**) are similar to those of the nongalled stems and the portions below the galls (**Figures 3B, C**), where the multiple sieve areas in sieve plates (**Figure 3C**) have evident callose deposition (**Figures 3D, E**). The gall proximal region (**Figure 3F**) has the vascular cambium differentiating phloem outward with no apparent reorganization, occupying the periphery of the structure (**Figure 3G**). The STE between parenchyma cells (**Figure 3H**), have callose deposition in the sieve areas (**Figure 3I**). The STE are connected to companion cells by sieve areas with small diameters compared with nongalled areas, which accumulate starch in chloroplasts and lipophilic substances (**Figures 3J, K**). The STE have numerous mitochondria and accumulate starch grains (**Figures 3K, L**).

**Figure 4:**
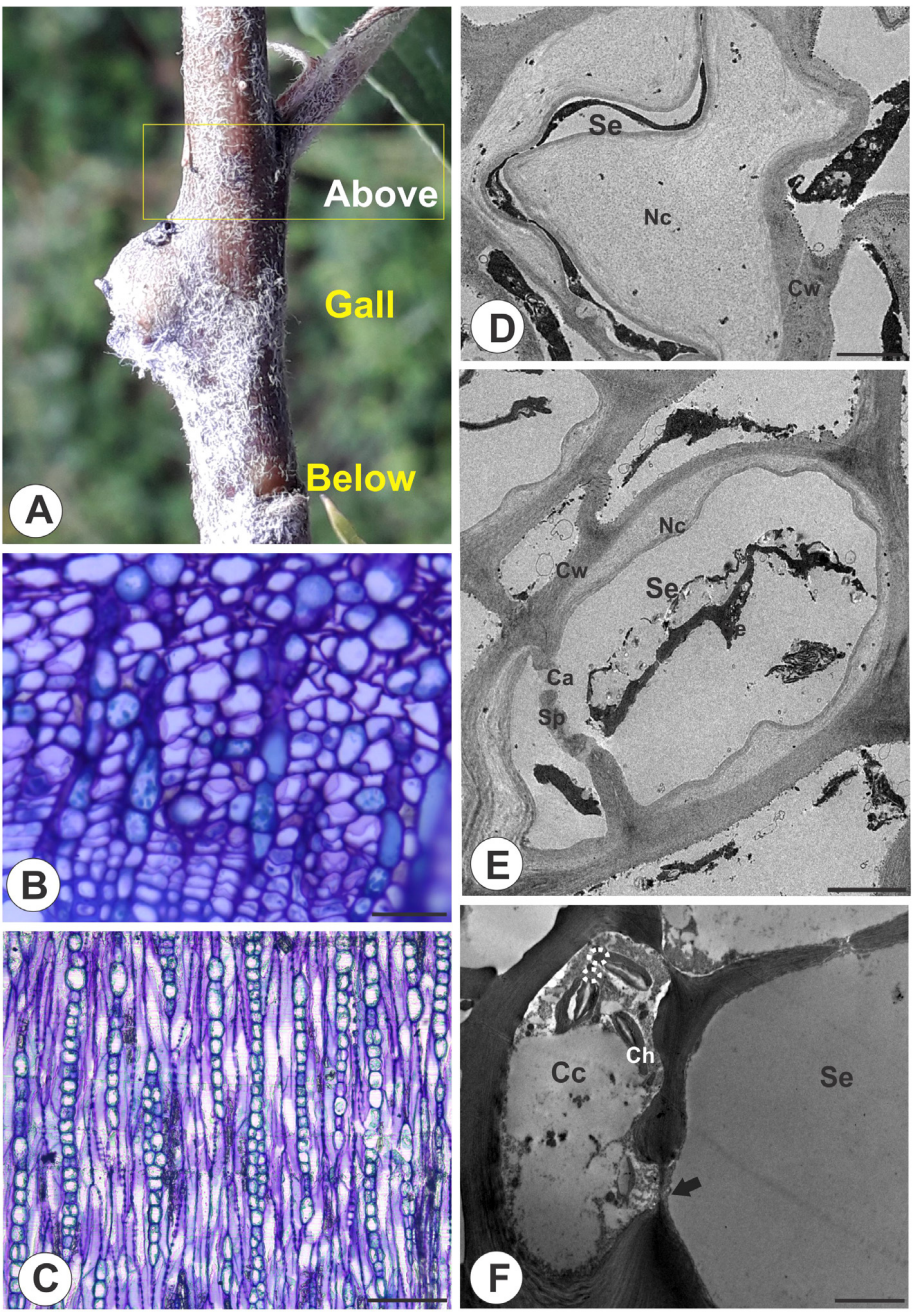
Stem portion above galls on *M. domestica*. **(A)** Stem portion evidencing the area above the gall. **(B)** Transverse section of the phloem portion showing the nacreous walls. **(C)** Longitudinal section showing the lateral sieve areas and sieve plates**. (D, F)** Transmission electron microscopy. **(D)** Sieve tube element lumen obliterated by nacreous walls. **(E)** Callose deposition in a sieve plate. **(F)** STE connected by sieve areas and plasmodesmata in degenerated companion cells. Se, sieve tube elements; Ca, callose; Cc, companion cells; Ch, chloroplasts; Cw, cell walls; Sp, sieve plate; Nc, nacreous. Bars: **(B, C)** 50 μm. **(D)** 2 μm. **(E, F)** 5 μm.

In the stem portions above the galls (**Figure 4A**), the phloem has anatomical characteristics similar to those of the stem portions below the galls and the distal region of the galls (**Figures 4B, C**), but the STE differ in terms of cell wall thickening. The STE lumen is reduced by these thickened nacreous particles (**Figure 4D**), and callose lines the pores of the sieve plates (**Figure 4E**). The STE are connected by sieve areas and plasmodesmata, and the companion cells are degraded (**Figure 4F**).

**Figure 5:**
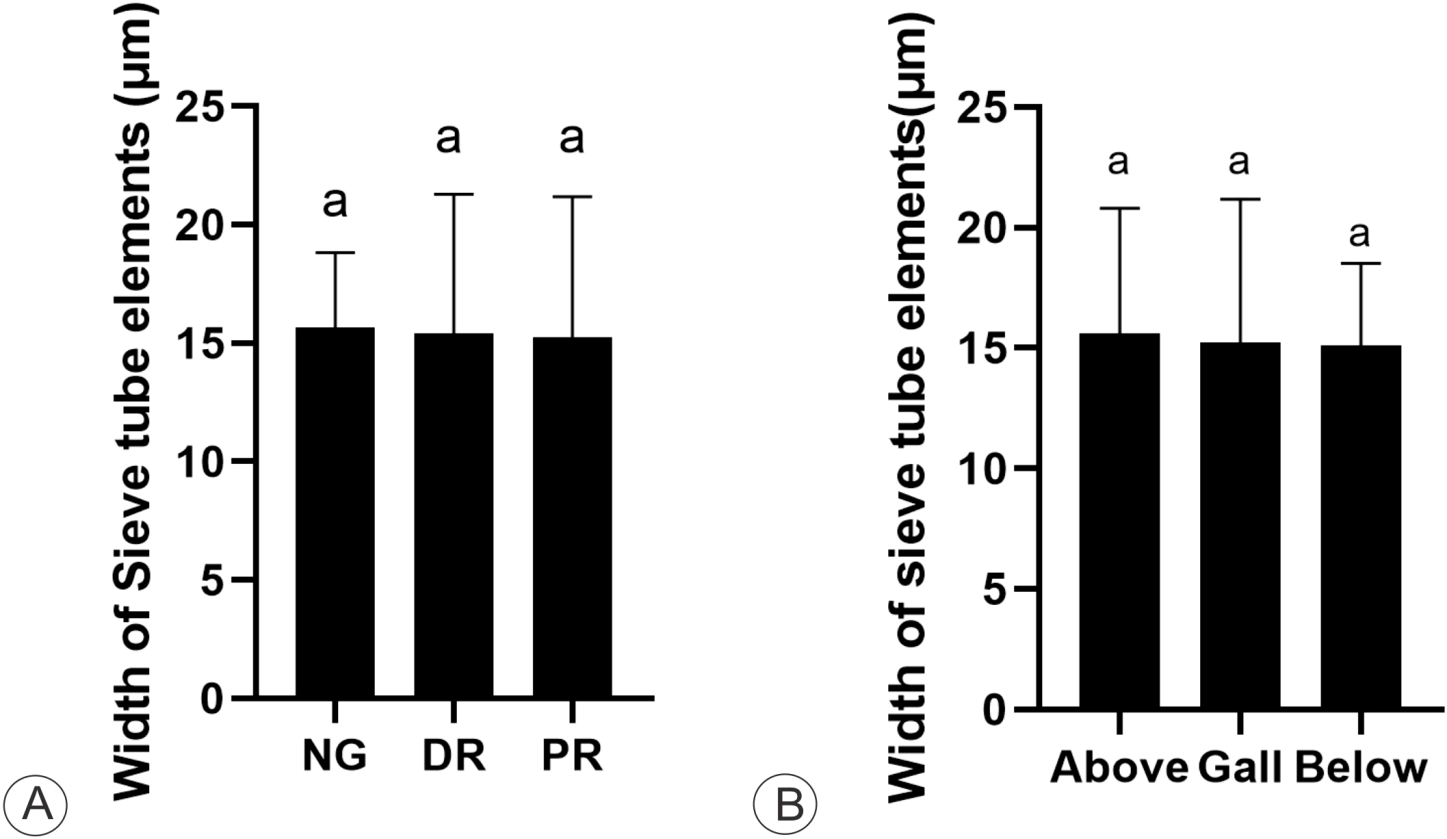
Cytometric analysis of sieve elements on *M. domestica*. **(A)** Mean width of sieve tube elements in non-galled stem (NG), gall distal (DR) and proximal regions (PR) (one-way ANOVA; Kruskal-Wallis). **(B)** Mean width of sieve tube elements of the stem gall (one-way ANOVA), and the stem portions above and below the gall.

The STE have similar dimensions among the galls, the nongalled stems, and the stem portions below and above the galls. The width of the STE in both nongalled stems and galls (proximal and distal regions) did not significantly differ (15.07 ± 3.2 μm) (p = 0.071) (**Figure 5**). The average dimensions of the STE did not differ significantly in width (p = 0.285) between the gall and the stem portion below the gall (15.1 ± 3.4 μm). Similarly, they are similar in width (p = 0.201) between the gall and the stem portions above the gall (15.4 ± 5.2 μm) (**Figure 5**).

## Discussion

The phloem cells in the non-galled stem portions and in the galls of *E. lanigerum* on *M. domestica* are similar, except for the evident thickening of the nacreous walls, decreasing the cell lumen area in the stem portions above the galls. Filamentous and granular P-proteins are characteristically observed just in the stem portions below the galls, closing the pores of the sieve plates. In cytological observations, the STE in stem galls maintain abundant starch accumulation, chloroplasts, and mitochondria, whereas the companion cells have a degenerated cytoplasm but with starch grains (Laitiainen et al., 2002). As conducting cells, the STE may have sealing systems to regulate and stop the flow of photoassimilates under stressful conditions, which can be performed by callose plugs (Eschrich, 1975). In *M. domestica* stem galls, the callose partially obliterates the STE in the distal region of the gall and stem portion above the gall, maintaining the cytoplasm of the STE, directing the flow of nutrients to the proximal region of the gall, favoring the feeding activity of *E. lanigerum.*

According to the gall constriction hypothesis, there is an increase in xylem differentiation with rays and vessel elements of regular size in stem portions below the gall. In contrast, the region above the gall has narrower vessel elements, increased rays, and an absence of fibers, which may limit the water transport to the aerial plant portion (Aloni et al., 1995). This hypothesis has not been corroborated in Neotropical host plant-gall inducer systems (Bragança et al., 2021; Jorge et al., 2022; Nobrega et al., 2023), which has made us focus on the phloematic portion of the vascular system and its implications to the flow of photoassimilates in galls. Accordingly, the variation in the dimensions of vascular cells in *M. domestica*, both for the xylem (Freitas, 2021) and the phloem, is not significant in the stem portions above and below the galls compared to the non-galled portions. Other cytological features in the gall portion relate to the high-water content necessary for the flow of photoassimilates in phloem conductive cells.

The transport of organic nutrients in plant organs may be reduced due to cytological peculiarities of the STE, such as the pectin-cellulosic wall composition (Kalmbach and Helariutta, 2019), as observed in *E. lanigerum* galls on *M. domestica*. In most plants, the STE wall thickening, described as a “nacreous” wall (Esau and Cheadle, 1958; Esau and Cronshaw, 1968; Nii et al., 1994; Knoblauch et al., 2020), can involve cellulose, pectins, and/or proteins variable deposition, turning the cell wall rigid. If the STE walls are rigid, the flow of photoassimilates would be directed toward the gall to the detriment of the non-galled stem portions, whose cell walls are thinner and more elastic. Being elastic, the STE expands radially if sap pressure increases, impairing the optimal flux of photoassimilates (Nakad et al., 2023).

Immunocytochemical analyses on the stem galls induced by *E. lanigerum* on *M. domestica* revealed the restricted composition of xylogalacturonans and xyloglucans in STE walls (Nogueira et al., 2024), i.e., they have a lower diversity of molecules than the phloem of non-galled stems. The nacreous walls in the STE of various Rosaceae fruits (Nii et al., 1994) decrease the area of the cell lumen, which seems to interfere with the free flow of photoassimilates (Esau and Cheadle, 1958), as observed in the stem portion above the *E. lanigerum*-induced galls on *M. domestica*. Such cytological features impose a higher pressure inside the STE in the galled regions. The active loading of sugars generates a low osmotic potential in the STE of the source, and consequently the ingress of water results in increased turgor (Patrick, 1997).

The alterations in phloem cells due to the interaction of *E. lanigerum colony* of *M. domestica* stems is not restricted to the STE. Some components of the protoplasts in the companion cells in stems and galls have a degenerated appearance, which has been also related to the nacreous wall formation in the fruit stalks of other Rosaceae (Nii et al., 1994). The companion cells degenerate when the associated sieve element becomes fragile and when components of the protoplast cannot be identified (Esau, 1973). In such a situation, the associated STE becomes inactive and may collapse or be completely obliterated (Esau et al., 1953), making the phloem nonfunctional. The companion cells are simplastically linked to sieve elements (Stadler et al., 2005) and connected at each end by sieve plates, which allow fluid flow and the passage of molecules, such as sugars and amino acids (Stadler et al., 2005; van Bel, 2018). In the case of apple trees, the sieve plates and pores vary in size, which represents different stages of development of the sieve plate, as seen in different species (Esau, 1939), and determine the conductivity of the sieve tube (Kalmbach and Helariutta, 2019), as is assumed to occur in *M. domestica*.

As expected, the sieve plates in the STE of the non-galled stems of *M. domestica* lack callose deposition, which can gradually accumulate over time, indicating the inactivation process of phloem transport (Aloni et al., 1991). The cell wall surrounding plasmodesmata is enriched with callose in the stem portions above the galls and the distal region of the stem galls on *M. domestica*, which can trigger a decrease in the flux of photoassimilates (Maule, 2008). In functional STE, callose deposition is common around the pores of sieve plates in lateral sieve areas and can be enhanced in response to diverse biotic and abiotic stresses (Radford et al., 1998). Together with callose, the filamentous and P-protein prevents the loss of assimilates (Ernst et al., 2012) and may close the pores of the sieve plates in some STE in the stem portions below the galls on *M. domestica*, demonstrating the ability to maintain the photoassimilates in the gall proximal region, favoring the pressure inside the local STE and, consequently, gall nutrition. Due to their hydrophobic nature, lipophilic substances are not expected in sieves tube, because the sap is an aqueous hydrophilic environment. However lipophilic substances in the STE of the portion below the gall and the proximal region may be involved in the interaction with proteins and facilitate their transport (Matilla, 2023). Many insect species use lipoprotein as metabolic fuel (Gilbert and Chino, 1974; Chino and Downer, 1982). Once facilitating transport, it is very pertinent to the feeding of *E. lanigerum* in the proximal region, since it can absorb other resources, such as amino acids, sugars, lipids, ions, and salts (Jing and Behmer, 2019).

The mitochondria in STE may increase from the non-galled stems to the stem galls in *M. domestica*, indicating high metabolism (McGivern, 1957; Hunt et al., 2023), and culminate in the accumulation of numerous starch grains in the phloem cells of both the non-galled stems and galls of *M. domestica.* Under natural conditions, starch accumulation is regulated by the sucrose level in the mesophyll, whose loading in phloem cells is determined by several metabolic pathways, involving synthesis, accumulation, and mobilization according to metabolic demands (Komor, 2000).

The intense feeding activity of the gall inducers may promote an increase in the number of phloem cells (Oliveira et al., 2006; Richardson et al., 2016). This increase is particularly notable with respect to the proliferation of STE and parenchyma in the proximal region of stem galls on *M. domestica*, which can contribute to the flow of assimilates. Additionally, the redifferentiation of the vessel elements and STE interspersed with this neoformed parenchyma may provide additional feeding sites for *E. lanigerum*, increasing the nutritional value of gall tissues.

## Conclusion

There were no differences in the dimensions of the STE in the gall and stem portions above and below the galls on *M. domestica*. Nevertheless, the increased deposition of callose in the stem portion above the gall and the distal region of the gall, the small pores in the sieve areas in the non-galled stem portions and the galls, and the nacreous walls decreasing the area of the lumen of the STE in the stem portion above the galls have functional implications. The increase in pressure inside the STE toward the gall proximal region favors the flux of photoassimilates in the feeding sites of the *E. lanigerum* colony but consequently affects the vigor of apple trees.

## Data Availability

The raw data supporting the conclusions of this article will be made available by the authors, without undue reservation.
